# The Antimicrobial Activity of Human Defensins at Physiological Non-Permeabilizing Concentrations Is Caused by the Inhibition of the Plasma Membrane H^+^-ATPases

**DOI:** 10.3390/ijms25137335

**Published:** 2024-07-04

**Authors:** María T. Andrés, Patricia Fierro, Victoria Antuña, José F. Fierro

**Affiliations:** 1 Laboratory of Oral Microbiology (LMO), University Clinic of Dentistry (CLUO), University of Oviedo, 33006 Oviedo, Asturias, Spain; andresmaria@uniovi.es (M.T.A.); patricia.fierro@scsalud.es (P.F.); uo282138@uniovi.es (V.A.); 2 Health Research Institute of the Principality of Asturias (ISPA), 33011 Oviedo, Spain; 3 SamerLabs SL, Asturias Technology Park, 33428 Llanera, Spain; 4 Primary Care Emergency Service, Cantabrian Health Service, 39000 Santander, Spain; 5 Deparment of Functional Biology (Microbiology), Faculty of Medicine, University of Oviedo, 33006 Oviedo, Spain

**Keywords:** human defensin, gamma-core motif, antimicrobial peptide, antimicrobial mechanism of action, H^+^-ATPase, Pma1p, HNP-1, HNP-4, hBD-2, hBD-3

## Abstract

Human defensins are cysteine-rich peptides (Cys-rich peptides) of the innate immune system. Defensins contain an ancestral structural motif (i.e., γ-core motif) associated with the antimicrobial activity of natural Cys-rich peptides. In this study, low concentrations of human α- and β-defensins showed microbicidal activity that was not associated with cell membrane permeabilization. The cell death pathway was similar to that previously described for human lactoferrin, also an immunoprotein containing a γ-core motif. The common features were (1) cell death not related to plasma membrane (PM) disruption, (2) the inhibition of microbicidal activity via extracellular potassium, (3) the influence of cellular respiration on microbicidal activity, and (4) the influence of intracellular pH on bactericidal activity. In addition, in yeast, we also observed (1) partial K^+^-efflux mediated via Tok1p K^+^-channels, (2) the essential role of mitochondrial ATP synthase in cell death, (3) the increment of intracellular ATP, (4) plasma membrane depolarization, and (5) the inhibition of external acidification mediated via PM Pma1p H^+^-ATPase. Similar features were also observed with BM2, an antifungal peptide that inhibits Pma1p H^+^-ATPase, showing that the above coincident characteristics were a consequence of PM H^+^-ATPase inhibition. These findings suggest, for the first time, that human defensins inhibit PM H^+^-ATPases at physiological concentrations, and that the subsequent cytosolic acidification is responsible for the in vitro microbicidal activity. This mechanism of action is shared with human lactoferrin and probably other antimicrobial peptides containing γ-core motifs.

## 1. Introduction

Mammalian defensins are a small group of Cys-rich cationic peptides synthesized via leukocytes and various epithelial cells that are effectors of innate immunity and modulators of the adaptive immune system [1,2,3,4]. In primates, defensins comprise three subfamilies (α-defensins, β-defensins, and θ-defensins) based on peptide size and the connectivity of six highly conserved cysteine residues forming three disulfide bonds [5,6,7]. However, only members of the α- and β-defensins groups have been found in humans, as determined using various molecular methods such as direct isolation from tissues or deductions via genetic analysis [8].

Despite significant progress in recent years, the structure–function relationship for human defensins is still poorly understood. In general, the antimicrobial activity of defensins is attributed to a disruptive effect on the cell membrane/s leading to the leakage of intracellular contents and subsequent cell death (Refs. [2,9], reviewed in [6,10]). These activities appear to be due to the interaction of the cationic regions of defensins with negatively charged phospholipids and lipopolysaccharide-containing membranes, which promote defensin binding to the cell membranes [11,12,13]. For example, the human α-defensins HNP-1 to HNP-4 induced the permeabilization of the outer and inner membranes of Gram-negative bacteria, associated with the loss of cell viability [14,15,16] and were also able to permeabilize artificial membranes [17,18]. Similarly, the candidacidal activity of human β-defensin hBD-2 was mediated via plasma membrane (PM) permeabilization [19] because of its ability to target the lipid phosphatidylinositol 4,5-biphosphate [13]. Interestingly, β-defensin hBD-3 exhibited a biphasic candidacidal activity, showing a permeabilizing effect only at relatively high peptide concentrations [20]. However, other lines of evidence suggest that defensin-induced microbial cell death is not necessarily related to membrane permeabilization, supporting the existence of other, as of yet unknown, mechanisms of antimicrobial action [21,22,23]. In support of this notion, it has been reported that the antimicrobial activity of some human defensins (i.e., HNP-1, hBD-2, and hBD-3) is dependent on the metabolic state of the target cell [24] and not related to membrane permeabilization [20]. These apparently contradictory results on the mechanism of action of human defensins may be due to (a) different experimental conditions and methods used, (b) structural differences in the cell wall of the microbial species or strains used, (c) the different cationic nature of the peptides, and/or (d) the peptide concentration used in the assays. In the latter case, it has been repeatedly reported that high peptide concentrations can lead to unspecific membrane permeabilization [18,20,25,26]. Since it is thought that membrane permeabilization is responsible for the antimicrobial activity of defensins both in vitro and under physiological conditions, numerous studies have been carried out on the interaction of these cationic peptides with lipid components of cellular or artificial membranes (Refs. [13,14,15,27], reviewed in [6,28]). However, experimental evidence suggests that other mechanisms of antimicrobial action may also be involved in vivo, and their characterization could provide a more precise understanding of the defensive function of defensins in both healthy and infected tissues, as well as allow for the development of new anti-infective therapeutic strategies. In this study, we assume that the mechanism of the antimicrobial action of natural host defense peptides should first be investigated in a cell-based system, under controlled restricted conditions using the lowest antimicrobial concentration of the peptide, since high concentrations could induce lethal effects mediated via mechanisms other than those occurring under physiological conditions.

The cysteine residues are part of the major determinants of the antimicrobial activity of all known antimicrobial Cys-rich peptides, including the large family of defensins [29]. In these peptides, multiple disulfide-bridged Cys residues conform to an ancestral 3D structure called γ-core motif. This structural motif is so closely associated with the antimicrobial activity of Cys-rich peptides that it even has predictive value for identifying previously unrecognized antimicrobial peptides [30]. We have previously shown that this archetypal structure also exists in some large human antimicrobial immunoproteins by identifying two γ-core motifs in the molecule of human lactoferrin and other members of the transferrin family of proteins [31,32,33]. Since human lactoferrin inhibits microbial H^+^-ATPases leading to cell death [34,35,36,37,38,39], the presence of the γ-core motif in the lactoferrin molecule suggested the involvement of this structure in the H^+^-ATPase inhibitory process [34,35]. This idea was supported by the striking similarities observed in the cell death pathway induced by human lactoferrin and kaliocin-1, a microbicidal short synthetic peptide containing a γ-core sequence of human lactoferrin [31,32,33,34].

The presence of a structural γ-core motif in human defensins prompted us to determine whether these host defense peptides could also inhibit microbial H^+^-ATPases. In this study, the above hypothesis was evaluated using a whole-cell-based system similar to that used to determine the inhibition of microbial H^+^-ATPases via lactoferrin [34,35] and other known H^+^-ATPase inhibitors [26,40,41]. For this purpose, representative members of the α-defensins (i.e., HNP-1, HNP-4) and β-defensins (i.e., hBD-2, hBD-3), shown in Table 1, were tested against two human aerobic pathogens, *Candida albicans* (yeast) and *Pseudomonas aeruginosa* (bacteria), to (a) determine the existence of a range of antimicrobial concentrations that do not permeabilize the plasma membrane; (b) determine the effects of different extracellular conditions and some cellular metabolism inhibitors or ion channel blockers on antimicrobial activity; and (c) compare the results obtained with those previously observed for human lactoferrin, a γ-core protein that inhibits microbial PM H^+^-ATPases (Refs. [34,35], reviewed in [39]).

Some effector peptides and proteins of human innate immunity contain archetypal structures associated with antimicrobial activity that have been conserved throughout evolution (e.g., γ-core motif). Unlike conventional antibiotics used in therapy, these peptides do not appear to have been selected for stable microbial resistance phenotypes after continuous coexistence with the human microbiome. Therefore, knowledge of the mechanisms of antimicrobial action of these immunity effectors and their function in different fluids and tissues of the human host may be useful for the development of future anti-infective therapies in the post-antibiotic era. Consistent with this idea, our study provides a new antimicrobial mechanism of action for human defensins by demonstrating that they are potent inhibitors of microbial PM H^+^-ATPase activity.

## 2. Results

### 2.1. Determination of Antimicrobial Non-Permeabilizing Concentrations of Human Defensins

**Figure 1:**
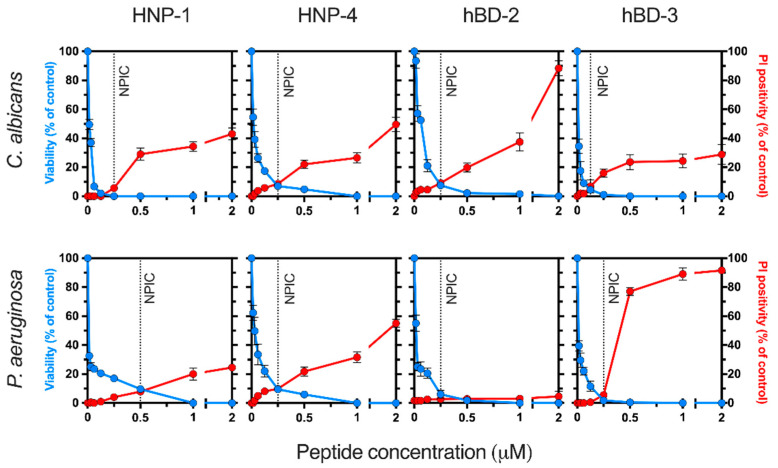
Antimicrobial and permeabilizing activities of human defensins. Microbicidal activity (blue lines) and membrane permeabilization (red lines) induced by defensins on *C. albicans* and *P. aeruginosa* cells. Cell suspensions (10^6^ cells/mL) in Tris buffer were incubated for 90 min at 37 °C with the defensins (0 to 2 μM). The percentage of surviving cells (viability) with respect to the untreated control (100%) was determined by counting colony-forming units. The effect of defensins on membrane integrity was monitored by determining the percentage of propidium iodide (PI)-positive cells. Dashed lines indicate the non-permeabilizing inhibitory concentration (NPIC) for each defensin, as defined in the Results Section. The results are the means ± SD from duplicates of three independent experiments.

The ability of antimicrobial concentrations of several human defensins (i.e., HNP-1, HNP-4, hBD-2, and hBD-3) to induce cell membrane destabilization was assessed. *C. albicans* and *P. aeruginosa* cells suspended in Tris buffer were incubated (90 min at 37 °C) with different concentrations (range 0–2 μM) of defensins. Under these experimental conditions, all peptides tested were microbicidal in a concentration-dependent manner (Figure 1). The highest candidacidal activity corresponded to HNP-1 and hBD-3, while hBD-2 and hBD-3 exhibited the most potent pseudomonacidal activity.

To determine whether the above microbicidal concentrations exert a permeabilizing effect on fungal and bacterial membranes, the influx of the fluorescent nucleic acid-binding dye propidium iodide (PI) in cells incubated with the peptides was assessed. In cells, dye uptake reflects membrane permeabilization, as PI itself cannot permeate through an intact phospholipid bilayer. Interestingly, the number of PI-fluorescent cells was not significantly increased after incubation (90 min at 37 °C) with low microbicidal concentrations (≤0.25 μM) of defensins, suggesting that the plasma membrane was not disturbed (Figure 1). Concentrations ≥ 0.25 μM induced an increase in intracellular PI in cells incubated with all defensins, except HNP-1, which showed permeabilizing activity on *P. aeruginosa* cells at concentrations ≥0.5 μM. These results indicated that the reduction in cellular viability (cells able to proliferate) via low concentrations of the human defensins tested (i.e., HNP-1, HNP-4, hBD-2, and hBD-3) did not involve membrane disruption, suggesting the existence of an alternative mechanism of action. To select an antimicrobial but non-permeabilizing concentration of each peptide for further assays, we defined as a non-permeabilizing inhibitory concentration (NPIC) the antimicrobial inhibitory concentration (≤10% viable cells) that permeabilized ≤ 10% of cells incubated (90 min at 37 °C) in buffer solution. The NPIC of each defensin corresponds to the microbicidal concentration calculated according to the data shown in Figure 1. These values for *C. albicans* were 0.25 µM for HNP-1, HNP-4, and hBD-2 and 0.125 for hBD-3. The NPICs for *P. aeruginosa* were 0.5 µM for HNP-1 and 0.25 µM for HNP-4, hBD-2, and hBD-3.

To determine whether defensins inhibit microbial H^+^-ATPases, cell death characteristics associated with the microbicidal activity of defensins (NPIC) were evaluated using a previously described cell-based method involving a series of metabolic inhibitors and different experimental conditions [26,34,35,36,41]. The following sections compare the killing pathways activated by human defensins and other PM H^+^-ATPase inhibitors.

### 2.2. Effect of Human Defensins on Cellular K^+^-Homeostasis

In previous reports, we have shown that human lactoferrin is not a membrane-permeabilizing protein but can induce a partial loss of intracellular potassium (K^+^-efflux) in yeast (mediated by the specific K^+^-channel Tok1p) as well as in bacteria [31,34,35,36,42,43,44]. Potassium efflux in lactoferrin-treated cells appears to be a homeostatic cellular response to the disruption of the proton gradient caused by the inhibition of the Pma1p H^+^-ATPase [36]. Furthermore, elevated extracellular K^+^ inhibits the lethal activity of lactoferrin [34,35,43]. To determine whether similar characteristics are associated with the antifungal activity of human defensins, the following assays were performed.

#### 2.2.1. Effect of High Extracellular K^+^-Concentration on Antimicrobial Activity

**Figure 2:**
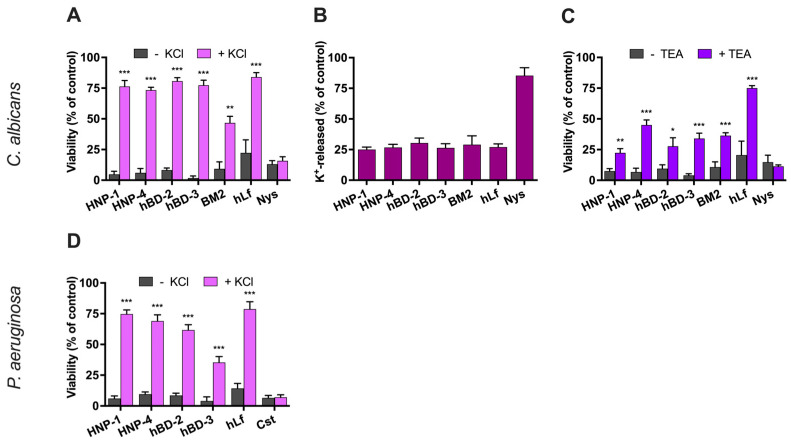
Effect of microbicidal activity of human defensins on cellular K^+^-homeostasis. (**A**) Effect of extracellular K^+^ on candidacidal activity. The candidacidal activity of defensins (NPIC) was tested using a cellular suspension of *C. albicans* in Tris buffer in the presence or absence of 50 mM KCl. (**B**) K^+^-efflux induced in *C. albicans* cells incubated with defensins. K^+^-release was measured after incubation (90 min, 37 °C) with defensins and compared with the total cellular K^+^-content (100%) of untreated cells. (**C**) Effect of inhibition of the K^+^-channel Tok1p on the candidacidal activity of defensins. Cells were preincubated for 15 min at 37 °C with 10 mM TEA (Tok1p inhibitor) before the addition of the peptides (NPIC). (**D**) Effect of extracellular K^+^ on bactericidal activity of defensins. The bactericidal activity of defensins (NPIC) was tested using a cell suspension of *P. aeruginosa* in Tris buffer in the presence or absence of 50 mM KCl. A plate colony-counting method was used to determine the percentage of cell viability relative to the untreated control (100%). Human lactoferrin (hLf) and BM2 peptide or nystatin (Nys) and colistin (Cst) were used as positive or negative controls, respectively. Error bars represent the SD of the means determined from three independent experiments. Student’s *t*-test: * *p* < 0.05; ** *p* < 0.01; *** *p* < 0.001 relative to the control.

Yeasts were significantly protected by the high external concentration of potassium (50 mM KCl) against the antimicrobial activity of defensins HNP-1, HNP-4, hBD-2, and hBD-3, used at their NPICs (Figure 2A). In control assays, the candidacidal activity of the Pma1p H^+^-ATPase inhibitors human lactoferrin and BM2 peptide [34,35,40] also significantly decreased (*p* < 0.01) in the presence of high extracellular K^+^-concentration. The pseudomonacidal activity of defensins and human lactoferrin (positive control) was also decreased in the presence of high extracellular potassium (Figure 2D). However, external potassium had no effect on the candidacidal activity of nystatin or the pseudomonacidal activity of colistin, two plasma membrane-permeabilizing agents used as negative controls.

#### 2.2.2. Evaluation of K^+^-Efflux Induced by Defensins

Defensins induced cellular K^+^-efflux with a similar pattern characterized by a partial loss of intracellular potassium ions. Potassium efflux in *C. albicans* cells occurred after cell exposure to these peptides (NPIC), reaching a steady state that was approximately four times lower than the maximum intracellular K^+^-concentration detected in untreated cells (Figure 2B). Similarly, microbicidal concentrations of human lactoferrin and BM2 peptide, both inhibitors of the fungal PM Pma1p H^+^-ATPase, induced a partial K^+^-efflux like that observed with defensins. In control assays, the membrane-permeabilizing peptide nystatin caused a high K^+^-release (approximately 70–85%) from yeast cells.

#### 2.2.3. Effect of the Inhibition of the K^+^-Channel Tok1p on Candidacidal Activity

In *C. albicans*, the K^+^-efflux induced by lactoferrin is mediated by the K^+^-channel Tok1p [36]. In fungi, Tok1p is a plasma membrane voltage-gated K^+^-channel with strong outward currents that are blocked by the tetraethylammonium cation (TEA^+^). Therefore, we wondered whether the fungicidal activity of defensins also requires the function of this specific potassium uniporter. The preincubation (15 min at 37 °C) of *C. albicans* cells with 10 mM TEA^+^ before the addition of defensins (NPIC) resulted in increased survival (Figure 2C). Similarly, the candidacidal activity of human lactoferrin and BM2 peptide (positive controls) was reduced in the presence of TEA^+^. As expected, the killing activity of nystatin (negative control) was not altered by this Tok1p inhibitor.

Taken together, these results confirm that the microbicidal activity of defensins at low concentrations is not related to a membrane-permeabilizing effect, and suggests the existence of a perturbation of cellular ion homeostasis, as previously reported for other PM H^+^-ATPase inhibitors (i.e., human lactoferrin, BM2 peptide).

### 2.3. Influence of Bioenergetics on the Microbicidal Activity of Defensins

The influence of some bioenergetic parameters on the microbicidal activity of low concentrations of defensins was studied and compared with those previously observed with lactoferrin.

#### 2.3.1. Influence of Cellular Respiration on the Microbicidal Activity of Defensins

*C. albicans* belongs to the metabolic group of yeasts known as Crabtree-negative species, meaning that it respires rather than ferments in the presence of low levels of glucose, and *P. aeruginosa* is an aerobic bacterium (not strictly speaking).

We previously demonstrated that human lactoferrin did not inhibit endogenous respiration in these microbial species but its microbicidal activity was significantly reduced in non-respiring cells [34,35]. Consistently, the oxygen consumption percentage of *C. albicans* exposed to defensins HNP-1 (87% ± 4%), HNP-4 (81% ± 3%), hBD-2 (89% ± 2%), and hBD-3 (79% ± 4%) after 30 min was not different from that observed in untreated cells (94% ± 2%) or in cells incubated with human lactoferrin (90% ± 3%), used as a negative control. However, yeast cells treated with 32 µM piericidin A (positive control), which inhibits the type I NADH dehydrogenase of the respiratory chain, showed low O_2_ consumption (20% ± 3%). Similarly, the respiratory function of *P. aeruginosa* cells was not altered by the presence of defensins as reflected in the O_2_ consumption values of HNP1 (72% ± 2%), HNP4 (75% ± 2%), hBD-2 (77% ± 3%), hBD-3 (82% ± 4%), or human lactoferrin (87% ± 2%) compared to untreated cells (83% ± 2%). As expected, 32 µM piericidin A, used as a positive control, reduced the oxygen consumption (17% ± 3%) of *P. aeruginosa* cell suspensions.

**Figure 3:**
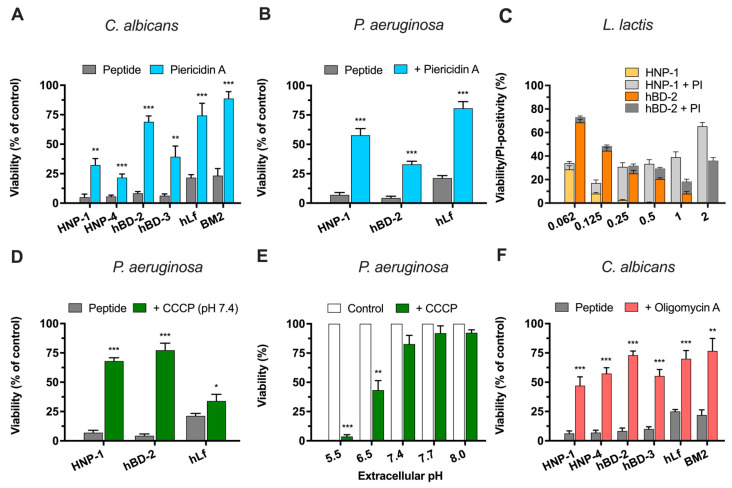
Influence of bioenergetic processes on the microbicidal activity of human defensins. (**A**) Viability of *C. albicans* cells treated with defensins (NPIC) in the presence of piericidin A (complex I inhibitor). (**B**) Viability of *P. aeruginosa* cells treated with the defensins HNP-1 or hBD-2 in the presence of piericidin A. In (**A**,**B**), cell suspensions (10^6^ cells/mL) in Tris buffer were preincubated (for 15 min at 37 °C) with the inhibitor and then incubated with the defensins (NPIC). (**C**) Viability of fermenting *L. lactis* cells treated with a range of concentrations of HNP-1 (yellow bars) or hBD-2 (orange bars). The effect of defensins on membrane integrity was monitored by determining the percentage of propidium iodide (PI)-positive cells after treatment with HNP-1 (grey bars) or hBD-2 (dark bars). (**D**) Viability of *P. aeruginosa* cells treated with defensin HNP-1 or hBD-2 (NPIC) after preincubation (for 15 min at 37 °C) with 50 μM CCCP (protonophore). (**E**) Viability of *P. aeruginosa* cells treated with 50 μM CCCP alone at different extracellular pHs. (**F**) Viability of *C. albicans* cells treated with the defensins HNP-1 or hBD-2 in the presence of oligomycin A (mitochondrial ATP synthase inhibitor). Human lactoferrin (hLf) or BM2 peptide was used as a positive control. The percentage of cell viability relative to the untreated control (100%) was determined using a plate colony-counting method. Data are from at least three similar experiments, and bars indicate ± SD. Student’s *t*-test: * *p* < 0.05; ** *p* < 0.01; *** *p* < 0.001.

To test whether the antifungal effect of defensins is influenced by active respiratory function, the killing effect was assessed using cells preincubated (15 min at 37 °C) with or without piericidin A. *C. albicans* cells treated with 32 µM piericidin A and exposed to defensins (NPIC) for 90 min were significantly less susceptible to HNP-1, HNP-4, hBD-2, and hBD-3 (Figure 3A). Similarly, *P. aeruginosa* cells pretreated with piericidin A were significantly (*p* < 0.001) protected from the bactericidal activity of the two defensins tested, HNP-1 and hBD-2 (Figure 3B). In control assays, the antimicrobial activities of human lactoferrin and BM2 peptide were also significantly (*p* < 0.001) inhibited by piericidin A.

In addition, we investigated whether the antimicrobial activity of human defensins also occurs under fermentative conditions. Killing assays were performed with *Lactococcus lactis*, a Gram-positive anaerobic homofermentative bacterium that switches to aerobic respiration only when heme, an essential cofactor of the terminal cytochrome *bd* oxidase complex, activates a short electron transport chain [45,46]. Under fermentative conditions, the *L. lactis* F-type ATPase operates in the ATP-hydrolyzing mode, pumping protons out of the cytosol using metabolic ATP as an energy source. Consequently, the inhibition of this enzyme complex results in the loss of proton-motive force (PMF) and cytosolic acidification, which can lead to cell death. *L. lactis* cells were exposed to two representative members of the α- and β-defensins (HNP-1 and hBD-2) in the range of 0.062 to 2 µM. The concentration-dependent bactericidal activity of both defensins and calculated NPIC (0.125 µM HNP-1 and 1 µM hBD-2) against fermenting bacteria is shown in Figure 3C. Human lactoferrin (0.062 µM) was also bactericidal against *L. lactis* (cell viability 13% ± 5%) in positive control assays, as previously reported [34].

Taken together, the above results show that (a) the electron transfer chain (ETC) components are not targeted by defensins and lactoferrin, as previously reported for human lactoferrin [34,35]; (b) the microbicidal activity of defensins occurs in both respiring and non-respiring microorganisms; and (c) the antimicrobial activity of defensins on respiring cells depends on a functional ETC. These features are shared by defensins with other H^+^-ATPase inhibitors, such as human lactoferrin and BM2 peptide, suggesting a common cell death pathway induced by a common cellular target (i.e., PM H^+^-ATPase).

#### 2.3.2. Effect of CCCP on the Defensin Bactericidal Activity

The protonophore carbonyl cyanide *m*-chlorophenylhydrazone (CCCP) combines with protons to form electroneutral CCCP molecules that passively diffuse across membranes, balancing the proton concentration on both sides of the membrane, and this constant H^+^ translocation cycle leads to the collapse of the proton-motive force (PMF or ∆*p*). The bactericidal activity of only two representative members of the human α- and β-defensins (HNP-1 and hBD-2) on *P. aeruginosa* cells was determined in the presence of CCCP. Figure 3D shows a significant reduction (*p* < 0.001) in antimicrobial activity when cells were preincubated (15 min at 37 °C) with 50 µM CCCP at pH 7.4. In control assays, the killing effect of lactoferrin on *P. aeruginosa* cells preincubated with CCCP was slightly reduced.

Protonophores also disturb intracellular pH homeostasis by promoting the equilibrium between intracellular and extracellular pH. To determine whether a change in intracellular pH could induce cell death, as reported for human lactoferrin [36], the viability of *P. aeruginosa* cells exposed to CCCP alone was determined. Figure 3E shows how the viability of *P. aeruginosa* decreased when cells were incubated with 50 µM CCCP alone at acidic extracellular pH (pH of 5.5 and 6.5). However, viability was not significantly altered when cells were incubated with the protonophore at alkaline pH (pH of 7.4, 7.7, and 8.0). This result shows that cytosolic acidification by itself induces a bactericidal effect, suggesting that situations that promote disturbed cellular ion homeostasis resulting in cytosolic proton accumulation can cause cell death. We have previously shown that human lactoferrin induces cytosolic acidification associated with cell death [36]. Because CCCP disrupts the mitochondrial proton electrochemical gradient in addition to that across the plasma membrane, we did not use this protonophore on *C. albicans* cells. However, it has been previously described that *C. albicans* cells treated with CCCP at pH 7.4 were protected against the candidacidal activity of HNP-1 [24,47] as well as hBD-2 and hBD-3 [20].

#### 2.3.3. Role of Mitochondrial ATP Synthase in the Candidacidal Activity of Defensins

Previously, we reported that the candidacidal activity of human lactoferrin was decreased in the presence of oligomycin A, an inhibitor of mitochondrial H^+^-ATPase [35,36]. *C. albicans* cells preincubated (15 min at 37 °C) with 16 μg/mL oligomycin A were significantly (*p* < 0.001) less susceptible to defensins HNP-1, HNP-4, hBD-2, and hBD-3 (Figure 3F). Similarly, the candidacidal activity of human lactoferrin and BM2 peptide (positive controls) was significantly reduced in oligomycin-treated cells, again suggesting a common cell death pathway for these Pma1p H^+^-ATPase inhibitors and human defensins.

### 2.4. Human Defensins Inhibit Fungal Pma1p H^+^-ATPase

In yeast, the PM Pma1p H^+^-ATPase is an ATP-dependent proton pump (ATP hydrolase) that generates a proton-motive force (PMF) across the cytoplasmic plasma membrane, consuming more than 50% of intracellular ATP, while acting as an important regulator of cytosolic pH [48]. Consequently, increased intracellular ATP levels, changes in electrical potential (Δφ), or decreased cellular H^+^-extrusion may reflect the altered functionality of the Pma1p H^+^-ATPase, and this triad of altered events has been proposed as a useful criterion in the search for new antifungal agents [26,41].

#### 2.4.1. Evaluation of Cellular ATP Levels in Relation to Candidacidal Activity of Defensins

**Figure 4:**
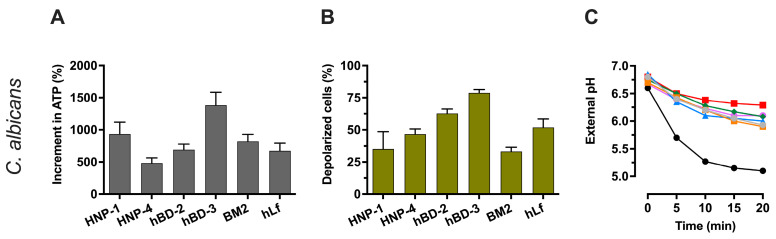
Effect of defensins on the total ATP level, the electrical potential of the plasma membrane, and the glucose-induced external acidification in *C. albicans*. Cells were preincubated (for 15 min at 37 °C) with defensins (NPIC) or with other peptides [5 μM human lactoferrin (hLf), 0.25 μM BM2 peptide] used as positive controls. (**A**) Increment in ATP levels in *C. albicans* cells exposed to defensins. The percentage change in total cellular ATP after 30 min treatment with the peptides (NPIC) compared to the untreated control is shown. (**B**) Relative level of membrane electrical potential of *C. albicans* treated with defensins. Percentage of DiBAC_4_(3)-positive cells (depolarized cells) exposed to defensins relative to untreated control (100%). (**C**) Effect of defensins on glucose-dependent external acidification. Glucose was added to induce the proton pumping activity of Pma1p H^+^-ATPase as indicated with external acidification in control assays. HNP-1 (grey line), HNP-4 (orange line), hBD-2 (blue line), hBD-3 (green line), BM2 peptide (violet line), human lactoferrin (red line), and control (black line). Data are from at least three similar experiments, and bars indicate ± SD. In (**C**), only the mean data (*n* = 3) are shown, and the bars representing the ±SD (coefficient of variation of ≤10%) are omitted for clarity.

The increase in cellular ATP levels in *C. albicans* cells after exposure to various Pma1p H^+^-ATPase inhibitors, such as human lactoferrin [35], BM2 peptide [26], or derivative carbazoles [41,49,50], has been reported previously. This ATP accumulation is attributed to the direct inhibition of the Pma1p-type H^+^-ATPase, which stops the consumption of ATP synthesized by the mitochondrial F-type H^+^-ATPase [26,48]. We observed an accumulation of ATP in response to exposure of yeast cells to defensins, consistent with a possible decrease in ATP consumption due to Pma1p H^+^-ATPase inhibition. Figure 4A shows increased ATP levels in cells exposed to NPIC of HNP-1 (~7-fold), HNP-4 (~8-fold), hBD-2 (~12-fold), and hBD-3 (~6-fold) compared to the untreated control. In agreement with previous reports, ATP levels were also increased with human lactoferrin (~6-fold) and BM2 peptide (~9-fold) used as positive controls [26,35]. These data are consistent with direct inhibition of the Pma1p H^+^-ATPase by human defensins.

#### 2.4.2. Effect of Defensins on The Electrical Potential of the Plasma Membrane

To further support the above view, we used the membrane potential-sensitive fluorescent dye bis-(1,3-dibutyl-barbituric acid) trimethine oxonol [DiBAC_4_(3)] to monitor the transmembrane electrical potential (∆φ) in *C. albicans* cells. DiBAC_4_(3) enters cells that have lost their membrane potential.

*C. albicans* cells treated with human defensins, as well as human lactoferrin or BM2 peptide (positive controls), resulted in an increase in DiBAC_4_(3) fluorescence, indicating a dissipation of ∆φ (Figure 4B). Since all these different peptides did not disrupt the plasma membrane at the concentrations used (NPIC), we conclude that the change in ∆φ is due to a perturbation of intracellular ion homeostasis as a result of previous Pma1p H^+^-ATPase inhibition, as reported for lactoferrin, BM2 peptide, and antifungal carbazoles [26,43].

#### 2.4.3. Effect of Defensins on H^+^-Extrusion by Pma1p H^+^-ATPase

A whole-cell proton transport assay was used to evaluate the possible inhibition of the fungal Pma1p H^+^-ATPase by defensins. In control assays (without peptides), starved *C. albicans* cells extruded protons via the Pma1p H^+^-ATPase after the addition of glucose (Figure 4C). This extracellular acidification was monitored with a pH electrode, which showed a progressive decrease of about 1.5 pH units from the initial value after 20 min (control). However, this external acidification was much lower in yeast preincubated with human defensins (approximately 0.8 pH units after 20 min). In positive control assays, cells preincubated with human lactoferrin or BM2 peptide showed similar inhibitions of H^+^-extrusion via the Pma1p H^+^-ATPase as previously reported [26,35,40].

## 3. Discussion

This study shows a novel antimicrobial mechanism of action of human defensins that is independent of their known membrane-disrupting activity. At low non-permeabilizing antimicrobial concentrations (≤0.25 μM), such as those found in human fluids [51,52,53], representative members of the human α- and β-defensins showed microbicidal activity, suggesting that these effectors of the innate immunity may exert their physiological function through a more sophisticated mechanism than previously known. Our results are in agreement with those of previous authors who suggested the existence of an alternative mechanism of action to the model of non-specific membrane destabilization previously proposed for defensins [20,21,22,23,54].

The presence of a γ-core pattern in the amino acid sequence of defensins [30] prompted us to evaluate whether these antimicrobial peptides can inhibit microbial H^+^-ATPases, ultimately leading to cell death in vitro. This idea was based on previous observations with human lactoferrin, another antimicrobial immunoprotein that inhibits H^+^-ATPases and contains two γ-core structural motifs [34,35,36,39]. To assess this hypothesis, we used a simple cell-based system with minimal elements and restricted experimental conditions [34,35]. The cell death induced by the human defensins shared several characteristics with the cell death pathway reported for human lactoferrin, as shown in Table 2. These common features were (1) cell death unrelated to plasma membrane perturbation, (2) the inhibition of microbicidal activity by extracellular K^+^, (3) the influence of cellular respiration on microbicidal activity, and 4) the influence of intracellular pH on bactericidal activity. In addition, in yeast, the following features were (1) the partial loss of intracellular K^+^ mediated by Tok1p K^+^-channels, (2) the essential role of mitochondrial ATP synthase in the cell death process, (3) intracellular ATP increase, (4) the depolarization of the cytoplasmic plasma membrane, and (5) the inhibition of external acidification mediated via PM Pma1p H^+^-ATPase. Some of these characteristics induced by human defensins and lactoferrin have been previously described for other different Pma1p H^+^-ATPase inhibitors, such as the synthetic antifungal peptide BM2 [26,40] and chemicals of the carbazole family [26,41,49,50]. Therefore, it can be suggested that human defensins, as well as all these structurally different agents, exert their microbicidal activity in vitro through a common cell death pathway related to the intracellular ionic imbalance caused by H^+^-ATPase inhibition. 

The loss of cytosolic ion homeostasis induced by human defensins in yeast is first supported by several observations, such as partial K^+^-efflux, membrane depolarization, and the involvement of the specific voltage-gated potassium channel Tok1p. We propose that this intracellular ion rearrangement is related to previous observations showing that active cellular metabolism is a prerequisite for the killing activity of some defensins, as also described for human lactoferrin [34,35], but that all these features are a consequence of the interaction of defensins with a specific membrane target. Previous investigators described that the active mitochondrial metabolism of the target cell is necessary to sensitize *C. albicans* cells to several human α- and β-defensins [20,24,47,55]. These authors suggested that cellular metabolism would maintain the membrane electrical potential necessary to electrostatically attract cationic defensins, or it could also indicate that the antimicrobial mechanism of some defensins is energy dependent [14,15,20,24,47]. However, based on our initial hypothesis and the experimental observations presented here, we have a new interpretation of these earlier proposals. Our data confirm all these previous results by showing that *C. albicans* and *P. aeruginosa* cells were protected from the lethal effects of defensins either when the respiratory chain was inhibited or when the proton-motive force (PMF) was collapsed through the action of the protonophore CCCP. However, defensins were highly active against *L. lactis* fermenting cells. Since *L. lactis* had no respiratory chain under these experimental conditions, it can be concluded that defensins do not require the specific presence of a functional respiratory chain for their antimicrobial activity and, therefore, these peptides do not act on any component of the ETC. On the other hand, the H^+^-ATPase of fermenting *L. lactis* functions as an ATPase (ATP hydrolase) that pumps protons from the cytosol at the expense of ATP and is essential for the generation of a PMF and pH regulation. Thus, our initial suggestion of an inhibition of this H^+^-ATPase by defensins is congruent with a possible accumulation of protons in the cytosol (cytosolic acidification) of *L. lactis* cells, leading to cell death. In this regard, we observed that CCCP alone was able to kill *P. aeruginosa* cells at acidic extracellular pH (e.g., pH 5.5) but not at physiological pH (e.g., pH 7.4). Assuming that PMF collapsed at both extracellular pH values (pH 5.5 and 7.4) and that intracellular pH equilibrated with extracellular pH, it can be concluded that the cause of death of cells exposed to acidic external pH (pH 5.5) in the presence of CCCP was due to cytosolic acidification. Thus, the fact that defensins did not kill CCCP-treated cells at pH 7.4 could be reinterpreted to mean that the protonophore removes excess protons accumulated in the cytosol, thus counteracting the lethal cytosolic acidification.

A surprising finding from our previous investigations with human lactoferrin was that the mitochondrial H^+^-ATPase activity of *C. albicans* cells was essential for the candidacidal activity of this γ-core protein [35,36]. Consistent with these prior studies, the candidacidal effect induced by human defensins was also significantly attenuated by oligomycin A, a specific inhibitor of mitochondrial F-type H^+^-ATPase, confirming the essential role of a functional ATP synthase for the progress of the defensin-induced cell death pathway. Taken together, this result and the aforementioned dependence on a functional respiratory chain in respiring *C. albicans* cells indicate the central role of mitochondria in the candidacidal activity of human defensins and lactoferrin [34,35,56], suggesting that both antimicrobials induce a common mitochondria-dependent cell death pathway [36]. Previous work with human lactoferrin has shown that this killing pathway results in apoptosis-like cell death [35,36,56].

Interestingly, we observed an increase in ATP levels in association with the candidacidal activity of defensins. This finding is in agreement with previous data on the fungicidal activity of HNP-1 on *C. albicans cells* [47]. A plausible explanation for this observation could be that the ATP was normally synthesized by the mitochondrial ATP synthase (F-type) but it cannot be simultaneously consumed by the plasma membrane Pma1p H^+^-ATPase (P-type) because it was inhibited by defensins. In addition to defensins, this unexpected effect was previously reported by us using lactoferrin-treated *C. albicans* cells [35], confirmed by Kjellerup et al. [26], and observed by different authors using other Pma1p inhibitors, such as the BM2 peptide [26] and various chemical agents from the carbazole family [41,49,50], as well as in *PMA1* mutants with an inefficient Pma1p H^+^-ATPase [57,58,59]. Thus, our result again supports that the inhibition of Pma1p H^+^-ATPase by human defensins, as well as lactoferrin, is the initial cause of their lethal effect in vitro.

Although it may seem paradoxical that cellular metabolism itself contributes to defensin-induced cell death, this apparent suicide makes sense when viewed solely from the perspective of cellular ion homeostasis. Therefore, and in the context of our proposal, we have recently suggested that the increase in cellular ATP means that a functional ATP synthase continuously synthesizes ATP molecules, but the required concomitant influx of protons into the mitochondrial matrix contributes to the alteration of mitochondrial ion homeostasis, ultimately leading to cell death [35,36]. Thus, the protective effect of oligomycin A in defensin-treated cells is not because this inhibitor prevents ATP synthesis, which could be necessary for a hypothetical energy-dependent antimicrobial mechanism, but because it prevents the entry of protons (via ATP synthase) into the mitochondrial matrix, which would otherwise result in a lethal alteration of mitochondrial ion homeostasis. These mitochondrial events, which have also been reported previously for human lactoferrin-treated cells, appear to be caused by a prior perturbation of cytosolic ion homeostasis induced through the inhibition of the PM Pma1p H^+^-ATPase [35,36]. All of the above results point to the alteration of the intracellular ionic environment as the main process responsible for the in vitro microbicidal activity of defensins.

Finally, to determine whether the Pma1p H^+^-ATPase is the target of defensins in the plasma membrane of whole cells of *C. albicans*, as the above experimental evidence seemed to indicate, we used a set of complementary assays that constitute a yeast-based drug discovery platform for the search of specific inhibitors of the Pma1p H^+^-ATPase. Kjellerup et al. [26] suggested that some common cellular events associated with Pma1p inhibition exerted by various antifungal drugs and observed by previous authors, such as the increase in intracellular ATP levels [26,35,41,57,59], the loss of transmembrane electrical potential [26], and the decrease of cellular proton extrusion [35,40,48], could together constitute a methodology to search for new antifungal agents targeting the PM Pma1p H^+^-ATPase. Consistently, ATP levels were increased as indicated above, and a loss of membrane potential and inhibition of Pma1p-mediated proton extrusion were observed in *C. albicans* cells treated with defensins. All these results indicate a direct inhibition of the fungal Pma1p H^+^-ATPase in intact cells and suggest that this essential component of the plasma membrane is a primary target of human defensins.

**Figure 5:**
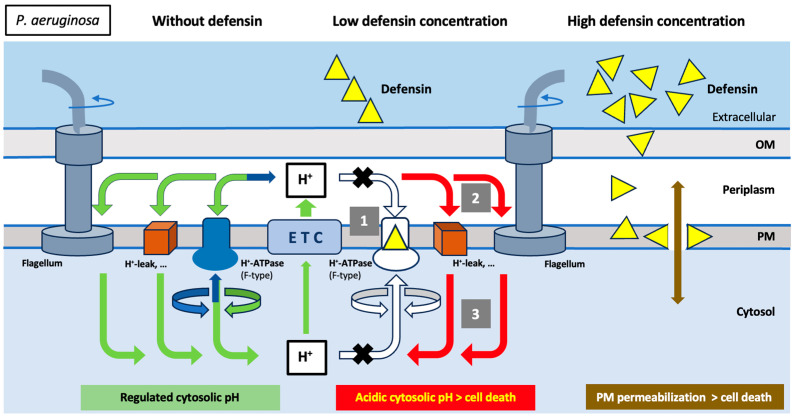
Schematic representation of the antibacterial mechanism of action proposed for the human defensins: Hypothetical defensin-induced cell death pathway in *P. aeruginosa* cells. The arrows indicate normal (green arrows), absent (white arrows), and involved (red arrows) ion fluxes. Involved ion fluxes would be part of the cell death pathway. The electrochemical proton gradient across the plasma membrane (PM) is generated by the electron transfer chain (ETC), and the re-entry of protons into the cytosol is used for various tasks, such as ATP synthesis (via F-type ATP synthase) or bacterial movement (via the flagellar rotor). Under these experimental conditions, the excess protons generated by active metabolism and gradually accumulating in the cytosol can be pumped out of the cell by the same F-type H^+^-ATPase, now operating in reverse mode and as an ATP-consuming ATP hydrolase (blue arrows). Defensins inhibit F-type H^+^-ATPase (1), preventing bidirectional proton flow through this functionally reversible H^+^-ATPase, thereby preventing intracellular pH regulation. Consequently, protons continuously pumped out of the cell via ETC activity hypothetically return to the cytosol (2) by various routes (flagellar rotor, H^+^-leak, etc.), but now they cannot be removed from the cytosol by reversion of H^+^-ATPase activity, and their progressive accumulation leads to lethal cytosolic acidification (3). This cell death pathway can be stopped at various levels through the use of appropriate inhibitors: high extracellular K^+^; piericidin A (inhibitor of type I NADH dehydrogenase of ETC); or CCCP, a protonophore that, at neutral or slightly alkaline extracellular pH, removes the excess protons accumulated in the cytosol and counteracts the lethal cytosolic acidification induced by defensin-mediated H^+^-ATPase inhibition. OM, outer membrane; PM, plasma membrane.

**Figure 6:**
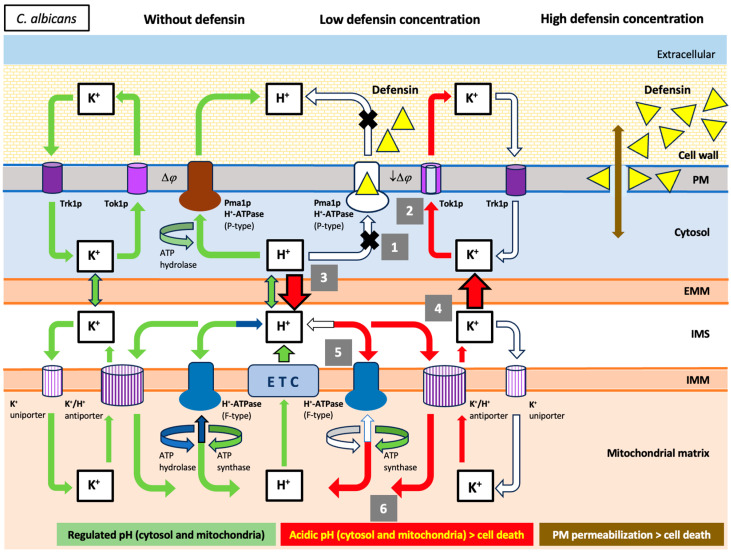
Schematic representation of the antimicrobial mechanism of action proposed for the human defensins: Hypothetical defensin-induced cell death pathway in *C. albicans* cells. The arrows indicate normal (green arrows), absent (white arrows), and involved (red arrows) ion fluxes. Involved ion fluxes would be part of the cell death pathway. In yeast, the Pma1p H^+^-ATPase unidirectionally pumps protons out of the cytoplasm to create a proton gradient across the plasma membrane (PM) and for internal pH homeostasis. Under these experimental conditions, the inhibitory effect of defensins on the Pma1p H^+^-ATPase appears to lethally perturb cellular ion homeostasis in two hypothetical concatenated phases. In the cytosolic phase, protons generated by active metabolism are accumulated in the cytosol due to the inhibitory effect of defensins on Pma1p H^+^-ATPase (1). The subsequent depolarization of the plasma membrane opens the voltage-gated potassium channel Tok1p, allowing a passive K^+^-efflux from the cytosol (2). These cytosolic events lead to the mitochondrial phase, in which the previous cytosolic acidification (3) and K^+^-efflux could facilitate a loss of mitochondrial potassium ions, favored by the simultaneous re-entry of protons into the mitochondrial matrix by the K^+^/H^+^ antiporter (hypothetical) and the F-type H^+^-ATPase (functions as an ATP synthase, as an increase in ATP was observed) (4) and (5). Eventually, the accumulation of protons in the mitochondrial matrix leads to acidification of this compartment (6), resulting in cell death. This cell death pathway can be stopped at various levels through the use of appropriate inhibitors: high extracellular K^+^-concentration [36], TEA (inhibitor of Tok1p K^+^-channel), piericidin A (inhibitor of type I NADH dehydrogenase of ETC), and oligomycin A (inhibitor of mitochondrial H^+^-ATPase). PM, plasma membrane; OMM, outer mitochondrial membrane; IMS, intermembrane space; IMM, inner mitochondrial membrane.

Taken together, the above results provide a new mechanistic understanding of the causal sequence of events culminating in microbial cell death induced by human defensins in vitro. The hypothetical defensin-induced cell death pathways shown in Figure 5 and Figure 6 are similar to those previously reported for human lactoferrin (Table 2) and support the idea of a common molecular target (i.e., PM H^+^-ATPase) for peptides and proteins containing a γ-core motif [34,35,36]. The inhibition of PM H^+^-ATPase appears to be the causal event leading to the intracellular acidification of the cytosol (bacteria) or mitochondrial matrix (yeast), which is ultimately responsible for the in vitro antimicrobial activity of defensins.

For the first time, in our knowledge, human defensins are shown to inhibit microbial plasma membrane H^+^-ATPases, as previously described for other γ-core proteins [34,35,36]. Since defensins are present at low concentrations in physiological fluids, this finding suggests that the inhibition of PM H^+^-ATPases is a physiological function of these effectors of the innate immune system. We encourage others to continue working to determine whether this mechanism of action (i.e., the inhibition of H^+^-ATPase) can be extrapolated to more defensins and the growing number of γ-core-containing peptides that are being discovered.

It appears that a limited number of ancient structural motifs present in effector molecules of innate immunity have been evolutionarily selected to interact with a few molecular targets of microorganisms that permanently colonize multicellular hosts. Antimicrobial peptides containing ancestral motifs rarely induce acquired resistance in microbiome and pathobiome components compared to conventional antibiotics, which may justify the evolutionary convergence and conservation of defensins along the Eukaryota and Bacteria domains [60,61]. Our findings highlight the importance of identifying other evolutionarily preserved archetypal motifs in immunoproteins to be used as stepping stones for the discovery of novel biological functions and therapeutic targets. The identification of these structures and the mechanistic understanding of their ancestral defense networks may be the model for a new therapy based on sustainable anti-infective strategies that avoid the selection of pathogen strains resistant to the antimicrobial agents used in clinical practice.

**Table 2:** Comparison of structural features and events associated with cell death pathways induced by some human antimicrobial γ-core peptides. Non-permeabilizing inhibitory concentration (NPIC) calculated for *C. albicans* (^a^) and *P. aeruginosa* (^b^) and other features found and/or confirmed in this study are shown in italics (references in superscript). Antimicrobial spectrum includes Gram-positive bacteria (Gm+), Gram-negative bacteria (Gm−), fungi (F), viruses (V), and protozoa (P). PM H^+^-ATPase, plasma membrane H^+^-ATPase; PM (V) H^+^-ATPase, plasma membrane (vacuolar) H^+^-ATPase; PM phospholipids, plasma membrane phospholipids; DNP, 2,4-Dinitrophenol; SHAM, salicyl hydroxamic acid; ND, not determined.

	Human α-Defensins		Human β-Defensins		Human Transferrins
	HNP-1	HNP-4	hBD-2	hBD-3	Human Lactoferrin
**Number of amino acids**	30	33	41	45	691 (protein)
**Archetypal antimicrobial structures** γ-core motif (number) Isomeric form (type)	Yes (one) Levomeric (2)	Yes (one) Levomeric (2)	Yes (one) Levomeric (2)	Yes (one) Levomeric (2)	Yes (two) Levomeric (1)
**Antimicrobial activity ** NPIC (μM) Cidal/static Membrane depolarization Membrane permeabilization Partial K^+^-efflux ATP increment/ATP-efflux Inhibition of H^+^-extrusion (yeast) Antimicrobial range	0.25 ^a^, 0.5 ^b^ Cidal [24,62] *Yes* [26] Yes [14] *Yes* *Yes* [47]/Yes [47] *Yes* Gm +,Gm −,F,V,P	0.25 ^a,b^ Cidal [62,63] *Yes* [12] Yes [23] *Yes* *Yes*/ND *Yes* Gm +,Gm −,F,V	0.25 ^a,b^ Cidal [20] *Yes* [11] Yes [11,13]/*No* [20] *Yes* *Yes*/ND *Yes* Gm +,Gm −,F,V	0.125 ^a^, 0.25 ^b^ Cidal [12,20] *Yes* [11] *No* [20]/Yes [11] *Yes* *Yes*/ND *Yes* Gm +,Gm −, F,V,P	5 ^a^, 1 ^b^ Cidal [34,35,64]/Static [65,66] *Yes* [43,67] No [31,34,42] *Yes* [31,42,43,44] *Yes* [35]/ND *Yes* Gm +,Gm −,F,V,P
**Cell death inhibitors ** *Extracellular inhibitors: * K^+^ or Na^+^ Ca^2+^ or Mg^2+^ Tok1p inhibitors (TEA) Anoxia *Intracellular inhibitors:* CCCP or DNP Azide Antimycin A (+SHAM) Piericidin A Oligomycin A	*Yes* [68] Yes (Ca^2+^)/No (Mg^2+^) [24] *Yes * Yes [24] *Yes* [24] Yes [20,24] Yes [24] *Yes* *Yes*	*Yes* [69] ND *Yes* ND ND ND ND ND ND	*Yes* [20] Yes [20] *Yes* ND *Yes * Yes [20] ND *Yes* *Yes*	No [11,12]/*Yes* [20] No [20] *Yes * ND ND Yes [20] ND ND ND	*Yes* [31,36,43] Yes [43] *Yes* [36,44] Yes [34,35] *Yes* [34] No [34] ND *Yes* [34,35] *Yes* [35]
**Cellular target**	* PM H^+^-ATPase *	* PM H^+^-ATPase *	*PM H^+^-ATPase * Phospholipids [13]	* PM H^+^-ATPase *	*PM H^+^-ATPase* [34,35,36] PM (V) H^+^-ATPase [39]
**Cell death type** (yeast)	ND Permeabilization [14]	ND Permeabilization [23]	ND Permeabilization (No [20]/Yes [11,13])	ND Permeabilization (No [20]/Yes [11,20])	Apoptosis-like cell death [36,44] No permeabilization [31,42]

## 4. Materials and Methods

### 4.1. Materials

Human defensins HNP-1, HNP-4, hBD-2, and hBD-3 (purity ≥ 99%) were purchased from PeptaNova GmbH (Sandhausen, Germany). BM2 peptide (_D_-NH_2_-RRRFWWFRRR-CONH_2_) was chemically synthesized and purified via high-performance liquid chromatography using GenScript (Piscataway, NJ, USA). Recombinant human apo-lactoferrin (hLf), colistin (polymyxin E), nystatin, oligomycin A, piericidin A, and tetraethylammonium (TEA) were purchased from Merck KGaA (Darmstadt, Germany). Bis-(1,3-dibutylbarbituric acid) trimethine oxonol [DiBAC_4_(3)], carbonyl cyanide *m*-chlorophenylhydrazone (CCCP), and propidium iodide (PI) were purchased from Thermo Fisher Scientific (Eugene, OR, USA). The BacTiter-Glo™ microbial cell viability assay kit was purchased from Promega (Madison, WI, USA). Sabouraud dextrose broth (SDB), Sabouraud dextrose agar (SDA), Tryptone Soya Broth (TSB), Tryptone Soya Agar (TSA), and M17 media were purchased from Oxoid Ltd. (Basingstoke, Hampshire, UK).

### 4.2. Strains and Growth Conditions

*Candida albicans* ATCC 10231 was obtained from the American Type Culture Collection (ATCC). *Pseudomonas aeruginosa* PAO1 and *Lactococcus lactis* subsp. *lactis* (strain IL1403) were kindly provided by Stephen Lory (Dept. of Microbiology, Harvard Medical School, Boston, MA, USA) and María-Cruz Martín (Instituto de Productos Lácteos, Villaviciosa, Asturias, Spain), respectively. For routine growth, cells were grown aerobically (150 rpm) in SDB at 30 °C (*C. albicans*), TSB (*P. aeruginosa*) at 37 °C, and M17 broth supplemented with 0.5% glucose (M17G) at 30 °C without shaking (*L. lactis*) for 16–20 h and subcultured in the same media until they reached the mid-logarithmic growth phase.

### 4.3. Antimicrobial Assays

Microbial susceptibility to the peptides was determined in a spread-plate assay as described previously [35]. Briefly, cell suspensions (10^6^ cells/mL) in Tris buffer (10 mM Tris-HCl, pH 7.4) were incubated for 90 min at 37 °C with 2-fold serial peptide dilutions. Peptide concentrations ranged from 0 to 2 μM. After incubation, the number of viable cells was determined by plating aliquots (100 μL) in duplicate on plates (SDA for *C. albicans*, TSA for *P. aeruginosa*, and M17G agarified medium for *L. lactis*) that had been dried for 30 min at room temperature in a laminar flow hood. Plates were incubated for 24–48 h at 30 °C (*C. albicans* and *L. lactis*) or 24 h at 37 °C (*P. aeruginosa*). Dilutions yielding 50–200 colonies were counted. Cell viability values, expressed as colony-forming units (CFUs), were calculated relative to the incubation without added peptides and represent the means ± standard deviation (SD) from duplicates of three independent experiments. For inhibition assays, unless otherwise noted, cell suspensions were preincubated (15 min at 37 °C) with the appropriate inhibitor prior to the addition of peptides, but the inhibitor was present throughout the incubation period with the peptide (90 min at 37 °C). Other specific experimental conditions are described as appropriate. In some experiments, the concentrations of human lactoferrin against *C. albicans* (5 μM), *P. aeruginosa* (1 μM), and *L. lactis* (0.065 μM) were used in the control assays. In various viability assays, BM2 peptide (0.25 μM), CCCP (50 μM), colistin (4 μg/mL), nystatin (50 μM), oligomycin A (16 μg/mL), piericidin A (32 μM), and TEA (10 mM) were used.

### 4.4. Permeability Assay

Flow cytometry was used to assess the ability of peptides to permeabilize cell plasma membranes, resulting in the incorporation of the DNA intercalating dye propidium iodide (PI). *C. albicans*, *P. aeruginosa*, and *L. lactis* cells (10^6^ cells/mL) in Tris buffer were incubated with two-fold serially diluted peptides for 90 min at 37 °C and then reincubated with PI (1 µg/mL, final concentration) for 5 min. Cell fluorescence was monitored using a CytoFLEX S flow cytometer (Beckman Coulter Life Sciences, IN, USA). The excitation wavelength was 638 nm and the emission at 780/60 nm. For each treatment, 5000 events were measured. Data were acquired and analyzed using CytExpert 2.1 Acquisition software. Results were expressed as a percentage of PI-positive cells relative to the unstained cells (control). The assays were performed with three independent experiments each conducted in duplicates.

### 4.5. Extracellular Potassium Measurement

Potassium measurements were performed via inductively coupled plasma mass spectrometry using an Agilent 7700x ICP-MS (Agilent Technologies, Santa Clara, CA, USA). *C. albicans* cells were cultured in SDB for 16–20 h at 30 °C. The cells were then subcultured in the same media to the mid-logarithmic growth phase and rapidly washed in sterile, deionized, double-distilled water. The cells (10^7^ cells/mL) were immediately incubated with the peptides (NPIC) for 90 min at 37 °C. The tubes were centrifuged at 1100× *g* for 10 min and the supernatants were collected and stored at 4 °C until analysis. The percentage of cytosolic potassium released from cell suspensions treated with nystatin (50 μM) was determined in positive control assays. Total cellular K^+^-content (100% value) was measured in the supernatant of cell suspensions previously treated with 0.5% (*v*/*v*) HClO_4_ alone, heated for 1 h at 95 °C, and centrifuged to remove cell debris. Results are expressed as the percentage (means ± SD) of K^+^-release relative to the total cellular K^+^-content from duplicates of at least three independent assays.

### 4.6. Oxygen Consumption Measurement

Oxygen consumption by *C. albicans* and *P. aeruginosa* cells was determined by polarographically using a biological oxygen monitor (dual digital-model 20; Rank Brothers Ltd., Cambridge, UK) at 25 °C. The apparatus consisted of a twin water-jacketed closed sample chamber, which enabled a control experiment to be conducted concurrently. The cells were grown to mid-log phase in SDB (*C. albicans*) or TSB (*P. aeruginosa*), washed twice in Tris buffer, and resuspended in the same buffer. Cell suspensions (approximately 10^7^ cells/mL) were preincubated with defensins (2 x NPIC), human lactoferrin (20 μM), or piericidin A (32 μM) for 15 min at 37 °C. Oxygen consumption was recorded at 5-min intervals for a period of 30 min. Three independent experiments were performed, and the data are means ± SD.

### 4.7. Measurement of ATP

Total ATP was measured using the BacTiter-Glo^TM^ Microbial Cell Viability Assay (Promega Co., Madison, WI, USA) according to the manufacturer’s instructions. Briefly, *C. albicans* cells were grown to mid-log phase in SDB at 30 °C, washed twice in Tris buffer, and resuspended at 10^6^ cells/mL. Aliquots (100 µL) were incubated (90 min at 37 °C) with human defensins (NPICs). Human lactoferrin (5 μM) or BM2 peptide (0.25 μM) was used as positive controls. One hundred microliters of the incubation mixture was transferred to an opaque 96-well plate, mixed with an equal volume of BacTiter-Glo^TM^ reagent, and incubated for 15 min in the dark. The reagent uses a thermostable luciferase to generate luminescence in an ATP-dependent manner. The emitted luminescence was detected using a Varioskan Flash multimode reader (Thermo Scientific, Waltham, MA, USA) with a 10 s shaking and 1 s integration time. A calibration curve was generated for each experiment with ATP standards ranging from 1 to 1000 nM. All assays were performed at least three times in duplicate.

### 4.8. Membrane Potential Monitoring

The membrane potential-sensitive probe DiBAC_4_(3) was used to monitor the transmembrane electrical potential of *C. albicans* cells as previously described [26] with slight modifications. Briefly, cells were harvested from mid-log phase cultures via centrifugation and suspended in Tris buffer. The cell suspension was adjusted to 10^6^ cells/mL and then incubated with DiBAC_4_(3) at a final concentration of 1 µg/mL for 30 min. Fluorescence was measured using a CytoFLEX S flow cytometer (Beckman Coulter) with excitation at 561 nm and emission at 690/50 nm. For each treatment, 5000 events were counted per experiment with an exposure time of 1000 ms. Results were analyzed using CytExpert 2.1 Acquisition software. As positive controls, human lactoferrin and BM2 peptide were used. Three independent experiments were performed and data are expressed as means ± SD.

### 4.9. Measurements of Acidification of the External Medium

To measure proton pumping from Pma1p H^+^-ATPase activity, a glucose-induced acid extrusion assay was performed as previously described [40,48] with modifications. Briefly, cultures of *C. albicans* cells were grown to mid-log phase in SDB medium and washed twice in 100 mM Tris-HCl (pH 8.0). The cells were then resuspended in 50 mM KCl and maintained for 18 h at 4 °C to reduce metabolic activity to a minimum (starvation conditions). To determine external acidification, starved cells (approximately 10^7^ cells/mL) were incubated for 15 min at 37 °C in the presence or absence of the defensins (2 x NPIC). Cells incubated with 20 µM human lactoferrin or 0.5 µM BM2 peptide were used as positive controls. The initial pH of the cell suspension was adjusted to approximately pH 6.8 with HCl, and glucose (final concentration of 2.5 mM) was immediately added to the cell suspension. The pH of the medium was recorded at 5-min intervals for 20 min using a SevenMulti S50-K pH meter (Mettler-Toledo, Greifensee, Switzerland) with a calibrated electrode (InLab 413, Mettler-Toledo) under constant stirring. Each experiment was repeated three times in an independent manner.

### 4.10. Statistics

Data analysis was performed using GraphPad Prism v.10.2.2 software (GraphPad Software Inc., San Diego, CA, USA). Student’s *t*-test was applied to data from independent assays to calculate significant differences between treated samples and untreated controls. Data showing statistical significance are indicated with asterisks: * *p* < 0.05, ** *p* < 0.01, and *** *p* < 0.001.

## Figures and Tables

**Table 1:** Amino acid sequence and physicochemical properties of human defensins used in this study. UniProtKB accession numbers are indicated in parentheses: HNP-1 (P59665), HNP-4 (P12838), hBD-2 (O15263), and hBD-3 (Q5U7J2). In yellow, the sequence γ-core of human defensins with the characteristic central triad of amino acids (Gly-X-Cys; levomeric isoform type 2) marked in grey (X means any amino acid). Conserved patterns of disulfide bridges between the Cys residues (in red) are shown. The complete sequence of the γ-core motif is shown (NH_2_… [C]-[X3–9]-[GXC]-[X1–3] …COOH) as originally defined by Yount and Yeaman [30]. Amino acids (number) [Aa (No.)], molecular mass (*m_av_*, average mass), net charge (z, at pH 7.0), and relative hydrophobicity (*H*_R_) were calculated using the Prot pi calculator (https://www.protpi.ch, accessed on 1 May 2024).

	Amino Acid Sequence	Aa (No.)	*m_av_*	*z*	*H*_R_
***α*-*defensins***	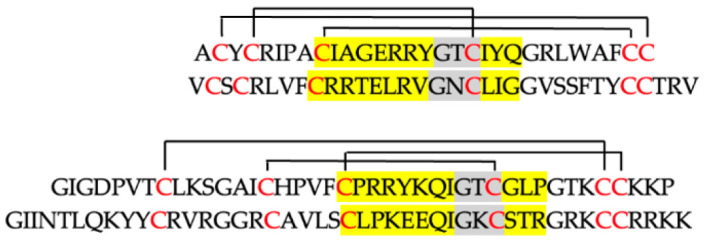				
HNP-1		30	3448	+3	38
HNP-4		33	3802	+4	39
***β*-*defensins***					
hBD-2		41	4328	+6	29
hBD-3		45	5155	+11	23

## Data Availability

All relevant data are within the manuscript and are available upon request to the corresponding author.
